# Association Analysis of a Microsatellite Repeat in the *TRIB1* Gene With Prostate Cancer Risk, Aggressiveness and Survival

**DOI:** 10.3389/fgene.2018.00428

**Published:** 2018-10-04

**Authors:** Leire Moya, John Lai, Andrea Hoffman, Srilakshmi Srinivasan, Janaththani Panchadsaram, Suzanne Chambers, Judith A. Clements, Jyotsna Batra, Australian Prostate Cancer BioResource, T. Yeadon

**Affiliations:** IHBIQUT, the Brisbane Urology Clinic, Aquesta Pathology, Sullivan Nicolaides Pathology; ^1^Australian Prostate Cancer Research Centre – Queensland, Translational Research Institute, Queensland University of Technology, Brisbane, QLD, Australia; ^2^Cancer Program, School of Biomedical Sciences, Institute of Health and Biomedical Innovation, Queensland University of Technology, Brisbane, QLD, Australia; ^3^Menzies Health Institute Queensland, Griffith University, Gold Coast, QLD, Australia; ^4^Cancer Research Centre, Cancer Council Queensland, Brisbane, QLD, Australia

**Keywords:** short tandem repeats (STRs), microsatellite, tribbles homologue 1 gene (*TRIB1*), prostate cancer, biomarker

## Abstract

With an estimated 1.1 million men worldwide diagnosed with prostate cancer yearly, effective and more specific biomarkers for early diagnosis could lead to better patient outcome. As such, novel genetic markers are sought for this purpose. The tribbles homologue 1 gene (*TRIB1*) has recently shown to have a role in prostate tumorigenesis and data-mining of prostate cancer expression data confirmed clinical significance of *TRIB1* in prostate cancer. For the first time, a polymorphic microsatellite in this gene was studied for its potential association with prostate cancer risk and aggressiveness. Genomic DNA was extracted from a cohort of 1,152 prostate cancer patients and 1,196 cancer-free controls and the TTTTG-*TRIB1* microsatellite was genotyped. The socio-demographic and clinical characteristics were analyzed using the non-parametric *t*-test and two-way ANOVA. Association of the TTTTG-*TRIB1* microsatellite and prostate cancer risk and aggressiveness were analyzed by binary logistic regression and confirmed by bootstrapping. Total and prostate cancer mortality was analyzed using the Kaplan Meier test. Genotype and allele correlation with TRIB1 mRNA levels was analyzed using the non-parametric Kolmogorov–Smirnov test. To predict the effect that the TTTTG-*TRIB1* polymorphisms had on the mRNA structure, the *in silico* RNA folding predictor tool, mfold, was used. By analyzing the publicly available data, we confirmed a significant over-expression of *TRIB1* in prostate cancer compared to other cancer types, and an over-expression in prostate cancerous tissue compared to adjacent benign. Three alleles (three–five repeats) were observed for TTTTG-*TRIB1*. The three-repeat allele was associated with prostate cancer risk at the allele (OR = 1.16; *P* = 0.044) and genotypic levels (OR = 1.70; *P* = 0.006) and this association was age-independent. The four-repeat allele was inversely associated with prosatet cancer risk (OR = 0.57; *P* < 0.0001). *TRIB1* expression was upregulated in tumors when compared to adjacent cancer-free tissue but was not allele specific. *In silico* analysis suggested that the TTTTG-*TRIB1* alleles may alter TRIB1 mRNA structure. In summary, the three-repeat allele was significantly associated with prostate cancer risk, suggesting a biomarker potential for this microsatellite to predict prostate cancer. Further studies are needed to elucidate the functional role of this microsatellite in regulating *TRIB1* expression, perhaps by affecting the TRIB1 mRNA structure and stability.

## Introduction

Australia and New Zealand, followed by North America, were the countries with the highest prostate cancer incidence in the developed world in 2012, according to the World Health Organization (Jemal et al., 2011). In 2016, 18,138 males were diagnosed with prostate cancer in Australia, accounting for 25.2% of all new male cancer cases and 12.8% of all male deaths caused by cancer (Welfare Aloha, 2016). In an increasing aging population, the numbers of newly diagnosed men with prostate cancer are likely to rise. Despite all the accumulated knowledge of the disease, the prostate-specific antigen (PSA) test remains one of the most common methods of screening since its introduction 30 years ago. However, the PSA test has gathered criticism for its potential over-diagnosis (Etzioni et al., 2002) and overtreatment consequences (Howrey et al., 2013). In 2012, The American Society of Clinical Oncology (Basch et al., 2012) and the U.S. Preventive Services Task Force (Moyer and U. S. Preventive Services Task Force, 2012) recommended against its use as a routine screening test. In this light, new and more specific biomarkers are needed.

Microsatellites, or short tandem repeats (STRs), are typically consecutive repeats of 2–5 base pairs (bp) of DNA in the genome. STRs are attractive biomarkers in human disease due to their highly polymorphic nature, abundancy, and wide distribution throughout the genome (Schlotterer, 2004; Willems et al., 2014). They have been used for high-resolution human genome mapping (Kong et al., 2002), population studies (Edwards et al., 1992; Budowle et al., 2002), and associated with up to 40 human monogenic diseases such as oculopharyngeal muscular dystrophy (Willems et al., 2014), as well as with more complex diseases such as cystic fibrosis and asthma (Hefferon et al., 2004; Batra et al., 2007).

Genome-Wide Association Studies (GWAS) genotype large cohorts of patients and controls, using high-throughput screening platforms to identify single-nucleotide polymorphisms (SNPs) that are associated with prostate cancer. This approach has been successful in identifying over 150 susceptibility loci for prostate cancer in a European cohort (Al Olama et al., 2014; Srinivasan et al., 2016; Schumacher et al., 2018). However, GWAS conducted to date can explain only up to ∼33% of prostate cancer heredity (Al Olama et al., 2014). We hypothesize that some of the missing prostate cancer genetic component can be explained by other genetic variants such as STRs. We used existing expression databases and bioinformatics tools that detect functional STRs lying in differentially expressed genes in prostate cancer as described previously by us (Lai et al., 2017), whereby we identified a penta-STR within the 3′UTR of the tribbles homologue 1 gene (*TRIB1*): TTTTG-*TRIB1*. Recent studies have associated the *TRIB1* gene with the development of several tumors including colorectal (Wang et al., 2017), leukemia (Yoshida et al., 2013), and hepatocellular (Ye et al., 2017) cancers. Also, in prostate cancer it has recently shown to have a role in cancer cell proliferation, survival and tumor growth (Mashima et al., 2014). Furthermore, independent clinical studies have reported higher expression of *TRIB1* in prostate cancer when compared to other cancers (Su et al., 2001; Ramaswamy et al., 2003) and an up-regulation of the *TRIB1* gene in prostate cancer tissue when compared to adjacent cancer-free cells (Liu et al., 2006; Grasso et al., 2012). However, the mechanisms by which its expression is upregulated in prostate cancer are not yet understood (Yu et al., 2004; Taylor et al., 2010; Grasso et al., 2012; Mashima et al., 2014). Additionally, we and others have observed a down-regulation of the TTTTG-*TRIB1* STR and the *TRIB1* gene (Munkley et al., 2015; Lai et al., 2017) in LNCaP cells after androgen treatment, supporting a link between *TRIB1* and prostate cancer. Interestingly, a recent study has observed an increase of *TRIB1* DNA copies and mRNA levels in breast cancer patients with a poorer survival outcome and a more aggressive phenotype in (Gendelman et al., 2017) after regulating proliferation, apoptosis, and cytokine production. Here, we undertook the largest genotyping analysis of a microsatellite repeat in this gene and analyzed its association with prostate cancer risk, aggressiveness and survival.

## Materials and Methods

### *TRIB1* Expression in Cancer

**FIGURE 1:**
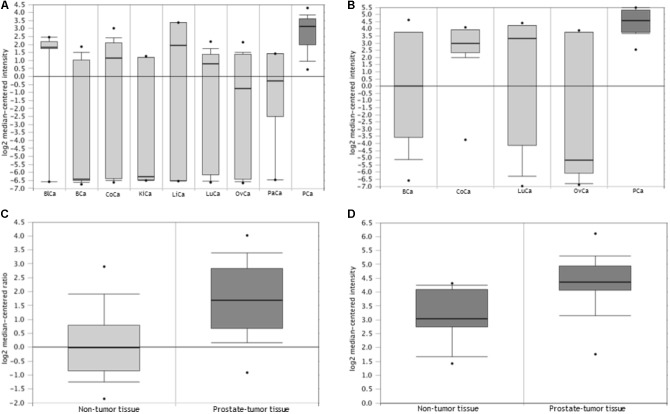
*TRIB1* is overexpressed in prostatate cancer. The Oncomine database showed that *TRIB1* was overexpressed in **(A)** prostate cancer when compared to eight (*n* = 174; Su et al., 2001), **(B)** and four other cancer types (*n* = 76; Ramaswamy et al., 2003) and in prostate cancer when compared to adjacent cancer-free tissue with in **(C)** Grasso et al. (2012) dataset (*n* = 122) and **(D)** Liu et al. (2006) dataset (*n* = 57); BlCa, bladder cancer; BCa, breast cancer; CoCa, colorectal cancer; KiCa, kidney cancer; LiCa, liver cancer; LuCa, lung cancer; OvCa, ovarian cancer; PaCa, pancreatic cancer; PCa, prostate cancer.

The Oncomine gene mining database (Rhodes et al., 2004) was used to determine how the *TRIB1* gene expression in prostate cancer compares with other cancers (**Figures 1A,B**) and to confirm if its expression was higher in prostate tumor tissue when compared to non-tumor prostatic tissue (**Figures 1C,D**). This mining web-tool extracts differential gene expression data from microarray analysis which originate from numerous samples such as cancer physiological fluids and tissues. Clinical and pathological data is also made available to the user. All the data presented is normalized and statistically analyzed.

In order to analyze the expression of the *TRIB1* gene in multiple cancer types, two multi-cancer studies were selected. The selection criteria were based on their number of samples and data availability for the prostate cancer. In the first study (**Figure 1A**) by Su et al. (2001) *TRIB1* gene expression data was available from prostate tumor as well as eight other cancers (*n* = 174; bladder, breast, colorectal, kidney, liver, lung, ovarian, and pancreatic). In the second study (**Figure 1B**), Ramaswamy et al. (2003) analyzed the *TRIB1* gene expression in prostate malignant tissue and compared it to four other cancer types (*n* = 76; breast, colorectal, lung and ovarian). The arrays used in the first study were the Human Genome U95A-Av2 Array while the HumanGeneFL Array, Hu35KsubA Array was used in the second study.

We then searched for reported differential expression of the *TRIB1* gene in prostate cancer when compared to prostatic benign tissue. Two studies that reflect this differential expression were selected for their number of samples available. The first study selected was carried out by Grasso et al. (2012; **Figure 1C**), where a total of 122 samples were analyzed (59 localized prostate carcinoma, and 28 benign prostate tissue specimens) using the Agilent Human Genome 44K array. The second selected study was presented by Liu et al. (2011; **Figure 1D**), where a total of 57 samples (44 prostate carcinoma and 13 adjacent normal samples) using the Human Genome U133A Array.

### Prostate Cancer Patients and Cancer-Free Controls

A total of 1,152 prostate patients were analyzed, including 138 men recruited via collaborations with urologists, 347 men from the QLD node of the Australian Prostate Cancer BioResource (APCB) and 667 men recruited in collaboration with the Cancer Council Queensland ProsCan study as detailed in our previous studies (Batra et al., 2011a; Lose et al., 2012; Eeles et al., 2013).

An extensive medical record for each patient that includes parameters such as age at diagnosis, family history of prostate cancer, ethnicity, PSA levels and survival data were collected, as well as pathology reports, including Gleason scores. Survival data was obtained through Cancer registry, which is maintained by Cancer Council Queensland (last extraction-2016). The registry operates under an Act of Parliament that requires mandatory notification of all cancer cases in Queensland by all hospitals (public, private, and psychiatric), nursing homes, and pathology laboratories. Death certificates are accessed to identify if the cause of death is cancer.

A total of 1,196 cancer-free control participants were included in the study. None of the controls included had been diagnosed with prostate cancer at the time of collection or presented any symptoms. From these, 527 men recruited through the Electoral Roll were age- and postal code-matched to patients from the ProsCan study. A further 669 male controls were recruited through the Australian Red Cross Blood Services. All cancer-free volunteers completed a detailed questionnaire that included information such as age, height and weight at the time of recruitment, and it also included risk factors and sociodemographic variables like family history of prostate cancer, whether they have had a vasectomy, as well as epidemiological factors such as, smoking, drinking and education. The scale used for all the variables are summarized in **Table 1**. All cases and controls were of European ancestry. This study was approved by the Queensland University of Technology’s Human Ethics Committee (Ethics’ Approval Number: 1000001171), and all participants provided informed written consent to participate in prostate cancer genetic studies.

**Table 1:** Socio-demographic and clinical characteristics of the Queensland study populations.

Characteristics	Men with prostate cancer (*n* = 1152) *n* (%)	Healthy controls (*n* = 1196) *n* (%)	*P-*values
Age in years (mean, range)	63.1 (40.2–87.1)	60.3 (18–89.6)	*P* < 0.0001^c^
BMI (mean, SD)	28.4 (4.7)	27.9 (4.5)	*P* = 0.091^c^
**Marital status**			
Never married	14 (1)	8 (1)	
Married/de facto	423 (37)	113 (9)	
Divorced/separated/widowed	44 (4)	13 (1)	*P* > 0.9^c^
Unknown	672 (58)	1063 (89)	
**Family history of prostate cancer^a^**			
No	498 (43)	796 (66.5)	
Yes	263 (23)	94 (8)	*P* > 0.9^d^
Unknown	392 (34)	306 (26)	
**Vasectomy status^b^**			
No	286 (25)	699 (58)	
Yes	148 (13)	445 (37)	*P* > 0.9^d^
Unknown	718 (62)	52 (4)	
**Smoking status**			
Never smoked	426 (37)	496 (41)	
Former smoker	582 (50.5)	580 (48.5)	*P* > 0.9^c^
Current smoker	78 (7)	83 (7)	
Unknown	67 (6)	37 (3)	
**Alcohol consumption^b^**			
Non-drinker	63 (5.5)	143 (12)	*P* > 0.9^d^
Drinker	370 (32)	1015 (85)	
Unknown	719 (62)	38 (3)	
**Highest education level achieved**			
No formal education	10 (1)	15 (1)	
Primary/Secondary school	516 (45)	469 (39)	*P* > 0.9^c^
Professional qualification	351 (30)	372 (31)	
University degree	211 (18)	304 (25)	
Unknown	62 (5)	37 (3)	
**Gleason score (Gleason grade 1 + Gleason grade 2)**			
<7	198 (17)	Not applicable	
≥7	785 (68)	Not applicable	
Unknown	171 (15)	Not applicable	

*^a^positive family history is defined as at least one first degree relative with prostate cancer. ^b^data was not collected for the retrospective study. ^c^P-values are from non-Parametric t-tests. ^d^Two-way ANOVA tests. body mass index (BMI) calculated as: kg/m^2^.*

### Genomic DNA Extraction and STR Genotyping

White blood cells were obtained from 10 ml of venous blood that were collected in EDTA tubes. Buffy coat was separated within 24 h and stored at -20°C until further processing. Genomic DNA was extracted from the buffy coats using the QIAamp DNA Mini Kit as per the manufacturer’s instructions (Qiagen, Hilden, Germany) as described previously (Batra et al., 2011b; Lose et al., 2012) and a multiplex PCR was performed in a 96 well plates. Briefly, the multiplex PCR kit (QIAGEN, Chadstone, VIC, Australia) was used in a 40 cycles’ PCR following the manufacturer’s guidelines. NED-fluorescently labeled primers (forward: 5′-GAGAAATGGCACAAAAACAGG-3′ and reverse: 5′-TTCTGTCAAGGTAATATTGCCAA-3′) were obtained from Sigma-Aldrich (Castle Hill, NSW, Australia). The primers were designed to amplify the STRs’ region at chromosome 8:126450287–126450311 position with a predicted product length of 299 bp (according to UCSC hg19). A total of thirty-one 96 well plates were subsequently analyzed according to their fragment sizes by capillary electrophoresis using the 3500 Genetic Analyzer platform (Applied Biosystems). Every plate had a well with water as a blank to determine the specificity of our PCR product and a total of 175 technical replicates were included across the plates to determine the reproducibility of the results. Technical replicates had to match in at least 90% of the hits detected to confirm the robustness of the assay. Due to the nature of the amplified and genotyped product, a 10% failure was allowed for potential mistakes at the PCR and/or genotypic levels. The results were then analyzed with GeneMapper v.5.0 (Thermo Fisher Scientific, Waltham, MA, United States).

### TTTTG-*TRIB1* RT-qPCR

The APCB prostate tumor bank provided formalin fixed and paraffin embedded (FFPE) blocks of prostate tissue, which contained both prostate tumor and adjacent non-tumor cells. The blocks were then sectioned sequentially in 20 μm sections and placed on glass slides which were methyl green stained. The pathologist marked tumor areas with their respective Gleason scores (**Supplementary Table 1**). mRNA was isolated using the RNeasy FFPE Kit (QIAGEN, Chadstone, VIC, Australia), mRNA was reversed transcribed (RT) using Superscript III (Life Technologies, Scoresby, VIC, Australia) as previously described (Lai et al., 2017). RNA purity and quantity were analyzed using the nanodrop^TM^ 2000/2000c Spectrophotometers and quantitative PCR (RT-qPCR) was performed using the SYBR Green method (Life Technologies, Scoresby, VIC, Australia). Twenty available patients’ tumor and adjacent non-tumor prostate tissue were selected based on their genotype as follows: 3/3 repeats (*n* = 2), 3/4 repeats (*n* = 8), and 4/4 repeats (*n* = 10). Their Gleason grades are detailed in **Supplementary Table 1**. The sequence at chr8:126450219-126450357 (UCSC hg19) was amplified with a predicted PCR product length of 139 bp using the forward, 5′-GAATGCCGTGTATACCTCACG-3′, and reverse, 5′-CGCAGGTTATTCAGACAGACA-3′ primers set. Applying the geometrical mean (geomean) of multiple housekeeping genes in RT-qPCR assists in removing non-specific gene variation expression, minimizing differences in the samples gene expression detection due to variables such as the RNA quantity and quality (Vandesompele et al., 2002). We therefore used geomean of the two control genes as reference, HPRT1 and RPL32. We then proceeded to evaluate the STR expression by calculating the ΔCT values = Ct TRIB1 – Ct Geomean of HPRT1 and RPL32.

### Statistical Analysis

After checking if age and body mass index (BMI) followed a Gaussian distribution, they were analyzed using a non-parametric, unpaired *t*-test (GraphPad Prism 7.00). For other parameters such as smoking, drinking, and marital status, their frequencies in both cases and controls were calculated and analyzed using a paired, non-parametric *t*-test. For those parameters where only two pairs of values were available such as family history, alcohol consumption and vasectomy status, a two-way ANOVA test was used. To test if the STR was within Hardy–Weinberg equilibrium (HWE), a chi-square test with a confidence level of 0.05 was used (Hwe, 2016). STR genotype and allele association with prostate cancer risk and aggressiveness analysis were performed using binary logistic regression and the odds ratios (ORs) were estimated as follows. For the allele analysis, the dependent variable was the prostate cancer status and the categorical covariate was the allele. In this analysis no reference was used as the covariate variable was either presence/absence of the allele (equal to 1 or 0, respectively). For the genotype analysis, the dependent variable was the prostate cancer status and the covariate variable was the genotypes. In here, the analysis used the major allele (4/4) as the reference. To verify that age was not biasing the results, a re-analysis was performed using the allele/genotype as the categorical covariate again, age as the second covariate and case-control status or Gleason score as the dependent variables. All tests were repeated using random sampling with replacement by bootstrapping analysis. The tests were seeded 1,000,000 times in 1,000 samples. Next, we analyzed the association of TTTTG*-TRIB1* STR with prostate cancer aggressiveness. A Gleason score of 8 and above is associated with a poorly differentiated or high-grade disease (Association of Directors of Anatomic and Surgical Pathology, 2018). We then grouped patients with a Gleason score of less than 8 and equal or greater than 8. Furthermore, since it has also been reported that the Gleason score pattern for a Gleason score of 7 can be an informative prognostic tool (Stark et al., 2009), where a higher primary Gleason score predicts a more lethal form of the disease, we then subdivided into 3+4 and 4+3 patients with a Gleason score = 7. Survival analysis was undertaken using the Kaplan Meier test [Log-Rank (Mantel-Cox)]. Data was plotted using GraphPad Prism 7.00. Hazard ratios (HR) were calculated using the Cox-Regression analysis for both total and prostate cancer related mortality. In this case the analysis was adjusted for age, Gleason score and PSA values. All analyses were conducted using IBM SPSS Statistics; 23.0 unless stated otherwise.

Differential *TRIB1* expression in prostate cancer tissue and adjacent normal tissue and correlation of genotype and allele data with STR mRNA levels were analyzed using the non-parametric Mann–Whitney test and plotted using GraphPad Prism 7.00. A two tailed *P* < 0.05 value was considered significant for all the analysis.

### TTTTG-*TRIB1* mRNA Structure Prediction

In order to assess the potential impact that the two most common alleles of TTTTG-*TRIB1* have on the TRIB1 mRNA folding structure, the *in silico* RNA folding predictor tool, mfold web server v.3^1^, was used (Zuker, 2003). There are two transcript variants of TRIB1 according to the RefSeq genes data set: transcript variant 1, NM_025195.3, and transcript variant 2, NM_025195.1. Both were analyzed with the two most common alleles, three- and four-TTTTG-*TRIB1* repeats. Since mfold calculates the most energetic favorable secondary structures that compose a mRNA molecule (given by their minimum free energy thermodynamic parameter, ΔG°), we hypothesized that the alleles may have an effect in the final folded structure and, potentially, in the final stability of the molecule, affecting the functionality and/or levels of the translated protein.

The Ingenuity Pathway Analysis (IPA; QIAGEN, Redwood City, CA, United States) was used to identify a list of miRNAs that bind to *TRIB1* and are deregulated in prostate cancer. From these, we sought for further confirmation in the miRNet database (Fan et al., 2016). Next, microRNA.org was used to find predicted miRNAs to bind near the STR (Betel et al., 2008). Finally, RNAhybrid (Rehmsmeier et al., 2004) was applied to detect if the miRNAs’ seed region of any of these miRNAs was altered by the allele change.

## Results

### *TRIB1* Is Highly Expressed in Prostate Cancer

Oncomine analysis (www.oncomine.org (Rhodes et al., 2004)) revealed *TRIB1* is highly expressed in prostate cancer when compared to other types of cancers in two multi-cancer clinical datasets, suggesting a specific role of *TRIB1* in this particular disease. Su et al. (2001) showed an overexpression (**Figure 1A**) in prostate cancer tissue when compared to bladder, breast, colorectal, kidney, liver, lung, ovarian, and pancreatic cancers (*n* = 174). Additionally, Ramaswamy et al. (2003) reported *TRIB1* to be overexpressed in prostate tumor tissue when compared to breast, colorectal, lung and ovarian cancer tissues (*n* = 76; **Figure 1B**). Furthermore, two independent data sets also showed an increase of mRNA expression in prostate cancer when compared to adjacent cancer-free tissue (**Figures 1C,D**; Liu et al., 2006; Grasso et al., 2012, respectively), which suggests that *TRIB1* may be associated with prostate cancer initiation and/or development.

### Demographic and Clinical Characteristics of the Cohort

Epidemiological data is shown in **Table 1**. The socio-demographic and clinical characteristics analysis showed no significant differences between cases and controls for all of the parameters analyzed (**Table 1**). Therefore, these parameters were not used further. Only the mean of the age was significantly different between the two groups (*P* < 0.05). This was expected due to the nature of the recruitment of some healthy controls, where samples from random blood donors were collected and some were younger participants than the patient cohort.

### The TTTTG*-TRIB1* STR Is Associated With Prostate Cancer Risk but Not With Aggressiveness

In some instances, one of the technical replicates failed, either due to amplification and/or electrophoresis issues. Of the genotyped duplicates, 92% showed concordant results, satisfying the quality control criteria of STR genotyping. We then proceed to analyze our STR genotyping results. Three alleles were observed for the TTTTG-*TRIB1* STR, varying from three- to five-repeats. All alleles were in HWE (*P* = 0.51). The three-repeat allele was associated with prostate cancer risk at the allelic (OR = 1.16; 95%; CI = 1–1.34; *P* = 0.044) and genotypic levels (OR = 1.70; 95% CI = 1.16–2.49; *P* = 0.006; **Table 2**). The four-repeat allele inversely associated with risk of prostate cancer at the allele level (OR = 0.57; 95% CI = 0.44–0.75; *P* < 0.0001; **Table 2**). Similar results were obtained after age adjusting and bootstrap reanalysis (**Table 2**). The rest of the genotypes (3/5, 4/5, and 5/5) were not analyzed due to the low number of observations (<1%).

**Table 2:** Allele and genotype risk association analysis of TTTTG*-TRIB1* STR with prostate cancer risk.

Genotype	Cases (%)	Controls (%)	OR (95%CI)^a^	*P*-value^a^	OR (95% CI)^b^	*P*-value^b^	*P*-value^c^	*P*-value^d^
3/3	74 (6)	47 (4)	1.70 (1.16–2.49)	0.006	1.65 (1.12–2.44)	0.011	0.006	0.01
3/4	365 (32)	384 (31)	1.03 (0.86–1.23)	0.75	–	–	–	–
3/5	1 (0.1)	0	–	–	–	–	–	–
4/4	701 (61)	759 (63)	1		–	–	–	–
4/5	9 (0.7)	9 (0.7)	–	–	–	–	–	–
5/5	2 (0.2)	0	–	–	–	–	–	–
**Allele**								
3	514 (22)	478 (20)	1.16 (1.00–1.34)	0.044	1.18 (1.01–1.36)	0.03	0.044	0.031
4	1776 (77)	1911 (80)	0.57 (0.44–0.75)	<0.0001	0.59 (0.45–0.77)	<0.0001	0.001	0.001
5	14 (0.6)	9 (0.4)	–	–	–	–	–	–

*Calculated using ^a^binary Logistic Regression, where the dependant variable is the case-control status and the covariate is the allele/genotype^b^age adjusted binary logistic regression, where age is the second covariate ^c^bootstrap (two-tailed) and ^d^bootstrap (two-tailed) age adjusted. (IBM SPSS Statistic Processor; 23). CI, confidence interval; OR, odds ratio.*

No significant association betweeen the genotypes and Gleason scores was obseved (*P* > 0.05; **Supplementary Table 2**), suggesting the TTTTG-*TRIB1* STR alleles have no effect in the disease’s aggressiveness.

**FIGURE 2:**
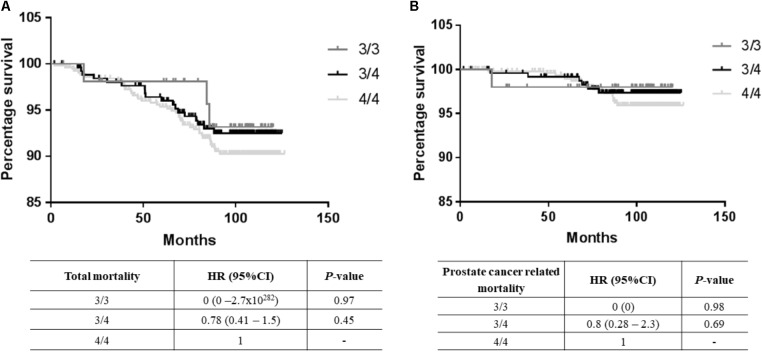
Survival data analysis. **(A)** Total mortality data [Log-Rank (Mantel-Cox) *P*-value = 0.16], **(B)** prostate cancer mortality data [Log-Rank (Mantel-Cox) *P*-value = 0.43] where TTTTG-*TRIB1* genotypes (3/3, 3/4, 4/4) are represented as a function of percentage survival over 125 months. HR and *P*-values were calculated using Cox-Regression analysis and adjusted for age, Gleason score and PSA values. HR, hazard ratio; CI, confidence interval.

A total of 67 deaths were reported from the total cohort of 1,152 patients, of which 25 were prostate cancer-related. No significant differences in mortality were observed between the 3/3, 3/4, and 4/4 TTTTG-*TRIB1* genotypes etiher for total mortality data [Log-Rank(Mantel-Cox) *P* = 0.695; **Figure 2A**] or prostate cancer related deaths [Log-Rank (Mantel-Cox) *P* = 0.466; **Figure 2B**] and these results were not affected by age, Gleason scores and PSA values (HR and their respective *P*-values are in **Figure 2**). Those patients with 5 TTTTG-*TRIB1* repeats (3/5, 4/5, and 5/5) were not included in the analysis due to their low incidence (*n* = 1, 3 and 7, respectively).

### *TRIB1* mRNA Expression Is Not Associated With the STR Allele

**FIGURE 3:**
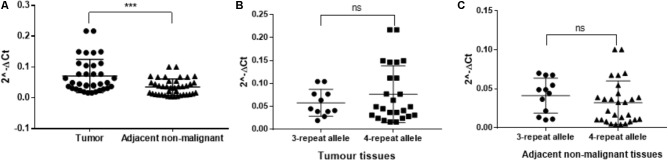
RT-qPCR from twenty patient tissue samples for TTTTG-*TRIB1*. Expression of the **(A)** *TRIB1* gene in tumor tissues when compared to adjacent non-malignant tissues and expression of the 3- and 4-repeats alleles in **(B)** tumor tissues and **(C)** adjacent non-malignant tissues. ^∗∗∗^*P* = 0.0005, value calculated using the Mann–Whitney test (GraphPad Prism 7.00).

The *TRIB1* gene expression showed a higher expression in prostate cancer tumor when compared to adjacent non-malignant tissue (*P* = 0.0005; **Figure 3A**). To identify if the STR alleles regulate *TR1BI* expression in tumor tissue, we analyzed the patient’s RNA from genotype two groups, i.e., carrying the three repeats allele or carrying the four repeats allele. No allele and TR1B1 mRNA expression correlation was observed either in the tumor (**Figure 3B**) or in the adjacent benign tissue (**Figure 3C**).

### TTTTG-*TRIB1* Alleles May Alter the mRNA Secondary Structure

**FIGURE 4:**
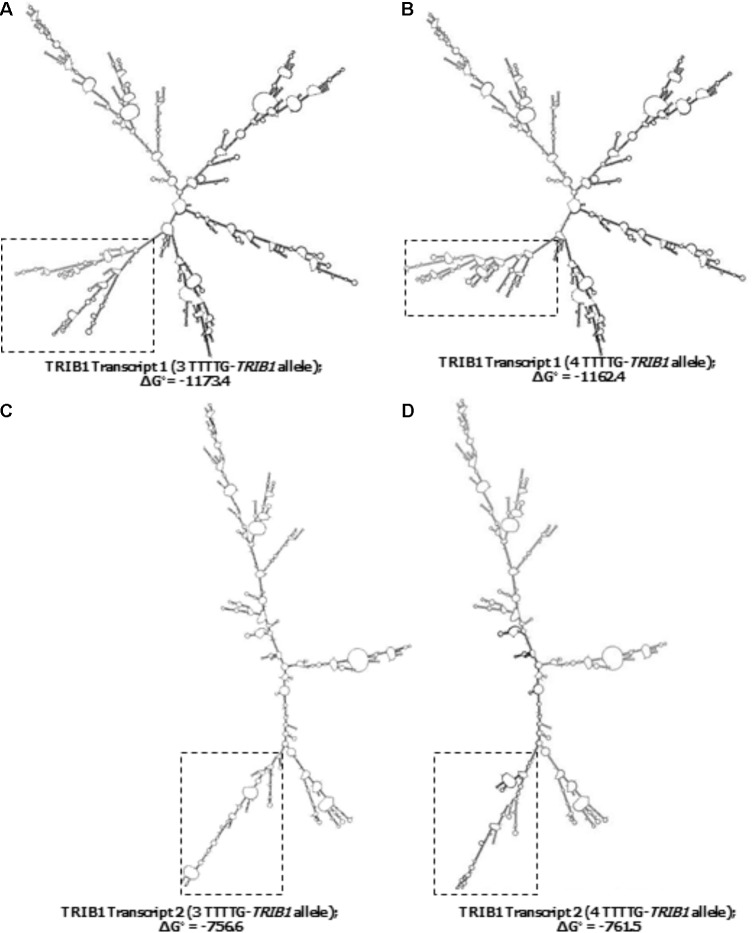
Predicted secondary structures of the two *TRIB1* mRNA transcripts. Showing two examples of the differences observed in the predicted TRIB1 mRNA structure of the *TRIB1* transcript variant one with the three **(A)** and four **(B)** TTTTG-*TRIB1* repeats alleles; and of the *TRIB1* transcript variant two with the three **(C)** and four **(D)** TTTTG-*TRIB1* repeats alleles. The dotted squares highlight the secondary structures differences in the final structure. Data obtained from mfold web server v.3 (http://www.bioinfo.rpi.edu/applications/mfold.).

From the 45–50 mRNA folded structures predicted by the *in silico* mfold tool, we focused on the top ten most energetically favorable ones, and compared how the change from three- to four-TTTTG repeats affected the structures of the two TRIB1 transcripts mentioned before. Four mRNA structures from the top ten were different as a consequence of the allelic change in the TRIB1 transcript variant 1 (NM_025195.3; example shown in **Figures 4A,B**). When the TRIB1 transcript variant 2 (NM_025195.1) was analyzed, there were eight mRNA predicted structures from the top ten that were different as a consequence of the allelic modification, including the most favorable structure (**Figures 4C,D**). This suggests the TTTTG-*TRIB1* alleles influence the mRNA folding structure and potentially this could also have an impact in the expression of the translated protein, which could explain the differences in prostate cancer risk observed in this study.

Since the TTTTG-*TRIB1* STR is within the 3′UTR, we investigated if the alleles have an effect in regulating the *TRIB1* gene expression by affecting the miRNA binding site region and interfering in the miRNA–mRNA duplex formation. The alleles of the TTTTG-*TRIB1* STR were not predicted to have an effect on any of the analyzed miRNAs binding.

## Discussion

Short tandem repeats are attractive biomarkers in human disease due to their highly polymorphic nature, abundancy and wide distribution throughout the genome (Schlotterer, 2004; Willems et al., 2014). They have been used for linkage mapping in several organisms, including humans (Weissenbach et al., 1992) and they have been associated with genetically simple and complex human diseases (Hefferon et al., 2004; Willems et al., 2014), including neurological disorders (Brouwer et al., 2009). A recent publication has shown massive STR sequencing (STR-Seq) is possible using the CRISPR-Cas9 technology (Shin et al., 2017). This study, published after our analysis was done, not only highlights the power of STRs as tools for genetic diagnosis, but it also shows the on-going interest of pursuing the sequencing of STRs for genetic association studies. However, this novel STR-Seq approach may not always be suitable to implement as it may depend on the resources available. In this study, we used a pre-screening approach where existing databases and bioinformatics tools were applied to detect STRs within differentially expressed genes in prostate cancer as shown in our recent publication (Lai et al., 2017). Using such methodology, we identified a penta-STR within the 3′UTR of the *TRIB1* gene: TTTTG. This gene is part of the tribbles family, comprised of three genes: *TRIB1*, *TRIB2*, and *TRIB3*. It is a highly conserved pseudokinase (Yokoyama and Nakamura, 2011) that lacks the protein-to-protein interaction domains and possibly any catalytic activity (Wei et al., 2012). Instead of directly phosphorylating proteins, this family of genes acts as an adaptor protein that regulates several cell pathways by enabling the degradation of targeted proteins after interacting with different cell mediators (Wei et al., 2012; Dugast et al., 2013). Intracellularly, *TRIB1* has an important role of regulating the C/EBP family of transcription factors (Yokoyama and Nakamura, 2011), responsible of processes such as transcription/translation and isoform formation (Hattori et al., 2003). *TRIB1* is also a known regulator of the mitogen-activated protein kinase (MAPK) pathway (Sung et al., 2007; Yokoyama and Nakamura, 2011), which activates/inhibits key cell processes such as growth, proliferation, differentiation, migration, and apoptosis, all of which have a pivotal role in cancer development and progression (Dhillon et al., 2007; Roberts and Der, 2007). In fact, *TRIB1* has been described as a myeloid oncogene due to its strong link with leukemia (Rothlisberger et al., 2007; Yokoyama and Nakamura, 2011). In a previously mentioned recent study, the *TRIB1* gene has also shown to play a significant role in prostate cancer, where its knockdown decreased the proliferation and survival of prostate cancer cells in a three-dimensional *in vitro* model, as well as it promoted prostate tumorigenesis in an *in vivo* xenograft model after gene over-expression (Mashima et al., 2014). The increase in prostate tumor growth observed was reversed in a *TRIB1* knockdown model. In addition, *TRIB1* has also been shown to be downregulated by androgens in LNCaP prostate cancer cells (Munkley et al., 2015; Lai et al., 2017). Since androgens are a key signaling molecule in prostate cancer development and the main current therapeutic target (androgen deprivation therapy), this suggests *TRIB1* plays a role in the onset and/or development of the disease. It is not surprising then that the publicly available database Oncomine (Rhodes et al., 2004), revealed numerous independent clinical datasets that showed an overexpression of this gene in prostate cancer tissue when compared to adjacent cancer-free cells (LaTulippe et al., 2002; Varambally et al., 2005; Liu et al., 2006; Taylor et al., 2010; Grasso et al., 2012). Interestingly, Oncomine analysis also showed that *TRIB1* is highly expressed in prostate cancer when compared to other types of cancers in two independent multi-cancer studies (Su et al., 2001; Ramaswamy et al., 2003, respectively). All this evidence supports a role of *TRIB1* in the onset and/or progression of the disease.

In this study, we genotyped over 2,000 patients and matching controls for the TTTTG-*TRIB1* STR and found three alleles (three, four, and five repeats) in this population. The three-repeat allele showed a prostate cancer risk association at both the allele (*P* = 0.044) and genotype levels (*P* = 0.006) and this was stronger in the genotype analysis (OR = 1.16 vs. OR = 1.70). This difference could be due to a synergistic effect of the two alleles when combined. This study also showed that the allele/genotype does not affect the disease aggressiveness. Both results showed not to be associated with age after being age-adjusted. Additionally, no association with prostate cancer survival was observed, possibly due to the low number of reported deaths in our data set. Further follow ups, where more prostate cancer related deaths may be reported, could elucidate the role of this STR as a prognostic tool if such role indeed exists. We did not observe age, Gleason score or PSA had an effect on survival analysis in our cohort despite Gleason scores have previously shown to influence the prognosis of prostate cancer patients (Wright et al., 2009). Although not significant, an opposite effect of the 4/4 genotype with survival compared to its risk association with prostate cancer was observed. This could be due to the context dependent function of these alleles in the tumor microenvironment of an aggressive cancer vs. less aggressive tumor conditions, which has also been reported in recent publications such as SNP association studies (Wiklund et al., 2009; Pomerantz et al., 2010, 2011; Ahn et al., 2011). For example, a risk allele of the MSMB gene has been reported to be associated with prostate cancer compared to controls, but its association was stronger for non-fatal prostate cancer. Furthermore, the frequency of the risk allele was higher for non-fatal prostate cancer compared with fatal prostate cancer, but it was also associated with a decreased rate of progression to prostate cancer specific mortality (Wiklund et al., 2009; Pomerantz et al., 2010). Ahn et al. (2011), also reported that a risk allele in the MSMB and 8q24 genes were associated with an increased risk of metastatic prostate cancer compared with controls but not with time of recurrence in prostate cancer cases following diagnosis. Therefore, the observation that that the 4/4 genotype individuals have poorer survival (although not significant) and it is inversely associated with risk of prostate cancer is not surprising. Another limitation of this study was that our access to prostate tumor tissue and their adjacent non-tumor cells was limited in number: *n* = 10 for the three-TTTTG repeats and *n* = 18 for the four-TTTTG repeats allele, and therefore the results should be read with caution. This could explain why no significant differences in allele specific mRNA expression were observed. Future analysis, where additional samples can be included may clarify if the *TRIB1* expression in prostate tumor tissue contributes to a poorer survival outcome as recently shown in a breast cancer study (Gendelman et al., 2017).

Since this STR is in the 3′UTR and its two most common alleles, three- and four-TTTTG repeats, have a difference of five nucleotides, we hypothesized this could affect either the miRNAs binding site or the final mRNA folding, and therefore have an effect in the stability and translation of the protein, potentially affecting the TRIB1 protein levels. Our miRNA analysis showed no differences in the 3′ UTR seed region when changing the STR allele from three- to four-repeats, suggesting the risk differences observed in this study are due to other regulatory mechanisms. Alternatively, there could be one or more miRNAs not yet identified that are affected by the polymorphism of the STR. We then focused on how the alleles could affect the TRIB1 mRNA structure. The predictor tool, mfold v3.0, showed the TTTTG-*TRIB1* alleles altered not only the order in which a given structure was most favorable but they also promoted unique structures for the two known TRIB1 transcripts. It would be interesting to investigate further the role of these two transcripts in prostate cancer development and if the TTTTG-*TRIB1* STR found herein has indeed an effect on TRIB1 mRNA stability, by possibly promoting or interfering in the translation of the mRNA. However, it is important to note that the minimum free energy method used by the mfold predictor tool has its own limitations such as assuming the RNA molecule is in equilibrium, that it has a single conformation and the nearest-neighbor effects are non-existent (Mathews, 2015). For these reasons, additional computational methods should be used to confirm the physiological significance of these findings and they also need to be experimentally validated by analyzing the allele specific protein expression of the *TR1BI* with a recently validated antibody (Soubeyrand et al., 2017). Finally, although we identified the TR1B1 STR through a method completely independent of GWAS, and for the first time analyzed the association of a polymorphism within the *TRIB1* gene, it must be mentioned that several SNPs associated with prostate cancer have been detected by GWAS in the same locus than the *TRIB1* gene (Gudmundsson et al., 2007, 2012; Srinivasan et al., 2016). It would therefore be imperative to establish the TR1B1 STR as an independent marker to GWAS identified SNPs at 8q24 for prostate cancer by undertaking the regression analysis conditioned on the GWAS loci.

In summary, the *TRIB1* gene is significantly overexpressed in prostate cancer when compared to other types of cancer and it is upregulated in prostate tumor tissue when compared to adjacent cancer-free cells. After genotyping over 2,000 prostate cancer patients and controls, we found the TTTTG-*TRIB1* STR is polymorphic and its three repeats allele has an association with prostate cancer risk at both the allelic and genotypic levels. However, no associations with mortality or aggressive disease were found. Further association studies in a larger cohort are warranted to confirm the outcome of our study. Our *in silico* predictions indicated that the TRIB1 mRNA structures are allele dependent. This repeat length could be affecting the mRNA stability and hence their expression level. Additional mechanisms by which this STR regulates *TRIB1* expression in an allele specific manner need to be further explored. Collectively, these findings validate our biomarker discovery approach methodology and highlight the utility of pursuing the use of STRs as biomarkers.

## Author Contributions

JL and JB conceived and designed the study and carried out bioinformatics analysis. LM and JB drafted the manuscript. LM, SS, and JP conducted the FFPE RNA extractions. LM and AH undertook the qRT-PCRs and Fragment analysis. LM and SS extracted DNA from patients’ blood samples. SC, JC, and the APCB provided the clinical samples. LM conducted the statistical analysis. JC provided vital feedback. JB supervised the study. All authors read and approved the final manuscript.

## Conflict of Interest Statement

The authors declare that the research was conducted in the absence of any commercial or financial relationships that could be construed as a potential conflict of interest.

## Footnotes

1 http://unafold.rna.albany.edu/?q=mfold/RNA-Folding-Form2.3
