# Supplementary material for: Association Analysis of a Microsatellite Repeat in the TRIB1 Gene With Prostate Cancer Risk, Aggressiveness and Survival
Source: Front Genet. 2018 Oct 4;9:428. doi: 10.3389/fgene.2018.00428 (PMC6180282; doi:10.3389/fgene.2018.00428)
Supplement: Supplementary file 1 [file Table_1.docx]

**Association analysis of a Microsatellite Repeat in the *TRIB1* Gene with Prostate Cancer Risk, Aggressiveness and Survival**

Leire Moya^1,2^, John Lai^1,2^, Andrea Hoffman^1,2^, Srilakshmi Srinivasan^1,2^, Janaththani Panchadsaram^1,2^, Suzanne Chambers^3,4^, Australian Prostate Cancer BioResource^2^, Judith A. Clements^1, 2^, Jyotsna Batra^1, 2^*.

^1^Australian Prostate Cancer Research Centre – Queensland, Translational Research Institute, Brisbane, Queensland, QLD, Australia.

^2^Cancer Program, School of Biomedical Sciences, Institute of Health and Biomedical Innovation, Queensland University of Technology, Brisbane, Queensland, QLD, Australia.

^3^ Menzies Health Institute Queensland, Griffith University, Gold Coast, Queensland, QLD, Australia.

^4^ Cancer Research Centre, Cancer Council Queensland, Brisbane, Queensland, QLD, Australia.

* **Correspondence**:

Associate Professor Jyotsna Batra

[jyotsna.batra@qut.edu.au](mailto:jyotsna.batra@qut.edu.au)

**Supplementary Table 1:** Prostate cancer tissue FFPE block Gleason grade (GG) and allele genotype.

| **Patient sample** | **Patient’ genotype** | **Tissue GG*** |
| --- | --- | --- |
| 1 | Homozygous (3 TTTTG-*TRIB1*) | 3 |
| 2 | Homozygous (3 TTTTG-*TRIB1*) | 3 |
| 3 | Heterozygous (3/4 TTTTG-*TRIB1*) | 3 |
| 4 | Heterozygous (3/4 TTTTG-*TRIB1*) | 3 |
| 5 | Heterozygous (3/4 TTTTG-*TRIB1*) | 3+4 |
| 6 | Heterozygous (3/4 TTTTG-*TRIB1*) | 4 |
| 7 | Heterozygous (3/4 TTTTG-*TRIB1*) | 3+4+5 |
| 8 | Heterozygous (3/4 TTTTG-*TRIB1*) | 3 |
| 9 | Heterozygous (3/4 TTTTG-*TRIB1*) | 3 |
| 10 | Heterozygous (3/4 TTTTG-*TRIB1*) | 3 |
| 11 | Homozygous (4 TTTTG-*TRIB1*) | 3 |
| 12 | Homozygous (4 TTTTG-*TRIB1*) | 3 |
| 13 | Homozygous (4 TTTTG-*TRIB1*) | 4 |
| 14 | Homozygous (4 TTTTG-*TRIB1*) | 4 |
| 15 | Homozygous (4 TTTTG-*TRIB1*) | 3 |
| 16 | Homozygous (4 TTTTG-*TRIB1*) | 4 |
| 17 | Homozygous (4 TTTTG-*TRIB1*) | 4 |
| 18 | Homozygous (4 TTTTG-*TRIB1*) | 3 |
| 19 | Homozygous (4 TTTTG-*TRIB1*) | 3 |
| 20 | Homozygous (4 TTTTG-*TRIB1*) | 3 |

*Marked by a pathologist in the FFPE slide where the RNA was extracted from
