# Supplementary material for: Association Analysis of a Microsatellite Repeat in the TRIB1 Gene With Prostate Cancer Risk, Aggressiveness and Survival
Source: Front Genet. 2018 Oct 4;9:428. doi: 10.3389/fgene.2018.00428 (PMC6180282; doi:10.3389/fgene.2018.00428)
Supplement: Supplementary file 2 [file Table_2.docx]

**Association analysis of a Microsatellite Repeat in the *TRIB1* Gene with Prostate Cancer Risk, Aggressiveness and Survival**

Leire Moya^1,2^, John Lai^1,2^, Andrea Hoffman^1,2^, Srilakshmi Srinivasan^1,2^, Janaththani Panchadsaram^1,2^, Suzanne Chambers^3,4^, Australian Prostate Cancer BioResource^2^, Judith A. Clements^1, 2^, Jyotsna Batra^1, 2^*.

^1^Australian Prostate Cancer Research Centre – Queensland, Translational Research Institute, Brisbane, Queensland, QLD, Australia.

^2^Cancer Program, School of Biomedical Sciences, Institute of Health and Biomedical Innovation, Queensland University of Technology, Brisbane, Queensland, QLD, Australia.

^3^ Menzies Health Institute Queensland, Griffith University, Gold Coast, Queensland, QLD, Australia.

^4^ Cancer Research Centre, Cancer Council Queensland, Brisbane, Queensland, QLD, Australia.

* **Correspondence**:

Associate Professor Jyotsna Batra

[jyotsna.batra@qut.edu.au](mailto:jyotsna.batra@qut.edu.au)

**Supplementary Table 2: Allele and genotype risk association analysis of *TTTG-TRIB1* STR with Gleason scores**

| **Genotype** | **GS < 8** | **GS ≥ 8** | **OR (95% CI)**^a^ | **p-value**^a^ | **GS = 3 + 4** | **GS = 4 + 3** | **OR (95% CI)**^a^ | **p-value**^a^ |
| --- | --- | --- | --- | --- | --- | --- | --- | --- |
| 3/3 | 54 (6) | 6 (5) | 0.72 (0.3 – 1.74) | 0.47 | 24 (5) | 16 (8) | 1.6 (0.82 – 3.1) | 0.17 |
| 3/4 | 268 (32) | 43 (33) | 1.04 (0.7 – 1.55) | 0.83 | 154 (33) | 58 (30) | 0.91 (0.62 - 1.31) | 0.6 |
| 3/5 | 0 | 1 (0.8) | - | - | 0 | 0 | - | - |
| 4/4 | 520 (61) | 80 (61) | - | Reference | 279 (61) | 116 (61) | - | Reference |
| 4/5 | 5 (0.6) | 1 (0.8) | - | - | 2 (0.4) | 1 (0.5) | - | - |
| 5/5 | 2 (0.2) | 0 | - | - | 2 (0.4) | 0 | - | - |
| **Allele** |  |  |  |  |  |  |  |  |
| 3 | 376 (22) | 56 (21) | 0.97 (0.70 – 1.34) | 0.86 | 202 (22) | 90 (24) | 1.07 (0.8 – 1.44) | 0.63 |
| 4 | 1313 (77) | 206 (78) | 1.31 (0.72 – 2.38) | 0.37 | 714 (77) | 291 (76) | 0.64 (0.4 – 1.01) | 0.06 |
| 5 | 9 (0.5) | 2 (0.8) | - | - | 6 (0.6) | 1 (0.3) | - | - |

Calculated using ^a^binary logistic regression, (IBM SPSS Statistic Processor; 23). GS: Gleason score; ns: no significant: CI: confidence interval
